# High exposure, rapid turnover: strain-level dynamics of *Klebsiella pneumoniae* carriage in community and hospitalized populations in Bangladesh

**DOI:** 10.1128/spectrum.01651-26

**Published:** 2026-08-25

**Authors:** Damien Slater, Sanchita Kar, Colin J. Worby, Jacquelyn Crowley, Zannat Kawser, S. M. Shamsuzzaman, Christian Harding, Ashlee M. Earl, Regina LaRocque, Firdausi Qadri, Jason Harris

**Affiliations:** 1 Division of Infectious Diseases, Massachusetts General Hospital 551672, Boston, Massachusetts, USA; 2 Massachusetts General Hospital for Children, Boston, Massachusetts, USA; 3 Harvard Medical School 1811 , Boston, Massachusetts, USA; 4 Institute for Developing Science and Health Initiatives, Dhaka, Bangladesh; 5 Infectious Disease and Microbiome Program, Broad Institute of MIT and Harvard 33577, Cambridge, Massachusetts, USA; 6 Dhaka Medical College and Hospital 468779, Dhaka, Bangladesh; 7 Omniose, Saint Louis, Missouri, USA; 8 Institutional Center for Diarrhoeal Disease Research 56291, Dhaka, Bangladesh; Universita degli Studi dell'Insubria, Varese, Italy

**Keywords:** *Klebsiella pneumoniae*, carriage, antibodies, antibiotic resistance

## Abstract

The gastrointestinal (GI) tract serves as a reservoir of *Klebsiella pneumoniae* (Kp), facilitating transmission of invasive strains and dissemination of antimicrobial resistance genes (ARG). Studies of drug-resistant strains in hospitalized patients, during care, and post-discharge, have shown that Kp may persist for months to years in the GI tract. In contrast, there are limited longitudinal data characterizing the duration of Kp GI carriage in healthy populations. In this study, we evaluated a previously described population from an urban neighborhood of Dhaka, Bangladesh, using metagenomic analyses of Kp-enriched fecal cultures to characterize strain-level and ARG carriage dynamics over a 2-month period, and we compared this with Kp carriage data from a contemporaneously enrolled hospitalized cohort from Dhaka Medical College Hospital. Our results show high rates of Kp carriage in both groups, along with frequent co-carriage of multiple drug-resistant strains. Overall, Kp strains were genetically diverse, and carriage of individual strains was typically short-lived, with extensive turnover between time points. We also observed age-associated increases in IgG antibody responses to common Kp surface antigens, which we postulate reflect the effect of repeated, frequent exposures to Kp in these populations. Together, these findings provide insights into the carriage dynamics of Kp in a highly endemic setting.

**IMPORTANCE:**

*Klebsiella pneumoniae* is a leading cause of antibiotic-resistant healthcare-associated infections, with the World Health Organization listing carbapenem-resistant strains as a priority pathogen. Gastrointestinal (GI) carriage is recognized as a risk factor for invasive disease in hospitalized patients, with the infecting strain often originating from the patient’s own gut microbiota. Less is known about non-hospitalized populations, where GI carriage is common but asymptomatic. By defining strain-level dynamics and characterizing the antimicrobial resistance genes (ARGs) circulating in a highly endemic community, this study provides a valuable counterpoint to previous research focused on hospital settings and contributes to our understanding of the diversity and dynamics of circulating strains in healthy populations where the burden of *Klebsiella pneumoniae* is high.

## INTRODUCTION

*Klebsiella pneumoniae* (Kp) is a Gram-negative opportunistic pathogen responsible for a spectrum of infections among immunocompromised and hospitalized individuals, including pneumonia, urinary tract infections, and bloodstream infections (1–3). Strains belonging to the classical Kp pathotype are associated with nosocomial infections, while hypervirulent Kp lineages can cause severe community-acquired infections, such as pyogenic liver abscess, meningitis, and disseminated disease in otherwise healthy individuals (4, 5).

Kp is also a major reservoir and disseminator of antibiotic resistance genes (ARGs). Multidrug-resistant (MDR) isolates producing extended-spectrum β-lactamases (ESBLs) and carbapenemases, including KPC, NDM, and OXA-type enzymes, have become increasingly prevalent worldwide (6–8). Kp’s genomic plasticity promotes horizontal transfer of resistance determinants across *Enterobacterales*, including important pathogens such as *Escherichia*, *Salmonella*, *Enterobacter*, and *Shigella* (9–11), and has facilitated the global spread of MDR clones such as ST258, ST307, and ST147 (12–14), earning Kp a place on the World Health Organization’s list of priority pathogens (15).

Asymptomatic gastrointestinal (GI) carriage serves as both a reservoir for transmission and a precursor to invasive infection, especially among hospitalized patients, where the infecting strain often originates from the gut (16–18). Carriage rates are elevated in hospitals and long-term care facilities and are associated with antibiotic exposure, older age, and comorbidities, such as diabetes, inflammatory bowel disease, and cancer (16, 17, 19). In the community, GI carriage rates vary widely, from ~5% in the USA and Australia to ~16% in Norway, and are substantially higher in Africa and Asia, reaching 40–66% among pregnant women and 19–88% among ethnic Chinese adults across multiple countries (16, 20–22).

The duration of Kp GI carriage remains poorly defined. Longitudinal studies in healthcare settings, primarily focused on carbapenemase-producing lineages, report persistence lasting months to years, particularly among patients with frequent healthcare exposure or chronic illness (23–25). Community-based investigations, mostly limited to ESBL- or carbapenemase-producing Kp or *Enterobacterales*, suggest that carriage of drug-resistant species may also persist for extended periods. However, the absence of strain-level characterization makes it unclear whether this reflects persistent or intermittent carriage with the same strain, or transfer of resistance determinants among different strains over time (26–28). One small Austrian study with strain-level resolution followed six healthy adults sampling weekly for one year and found only five instances where the same strain was found in two consecutive weeks, suggesting that in this population, Kp GI carriage is largely transient (29).

To address these gaps, we investigated Kp GI carriage prevalence, duration, and immunologic correlates in community and hospitalized cohorts in Dhaka, Bangladesh, a highly endemic region for antimicrobial resistance. This study builds on a prior 2-month study of healthy individuals, in which 75% (57/76) of participants had Kp detected at multiple time points (30). Here, we performed strain-level analyses of colonization dynamics and compared patterns with a contemporaneous hospitalized cohort. We also quantified serum antibody responses to Kp surface antigens to assess links between host immunity and carriage duration across age groups.

## RESULTS

### Study population

The population evaluated in this study was comprised of two groups: a community cohort that was reported on previously (30) and a new cohort of hospitalized adults recruited from Dhaka Medical College Hospital, Bangladesh.

The community cohort consisted of 75 individuals without major medical co-morbidities recruited from an urban neighborhood of Dhaka, Bangladesh, including 25 children under 5 years of age, 24 children aged 5–17 years, and 26 adults ages ≥18 years. Stool and blood samples were collected from all individuals at four time points over a 2-month period (day 0, 7, 30, and 60). In the present study, we extend the analysis of this cohort using metagenomic approaches to characterize circulating *K. pneumoniae* species complex (hereafter referred to as Kp) strains and antimicrobial resistance genes and to measure serum antibody responses to Kp antigens in the population.

To compare Kp carriage between healthy and hospitalized individuals, we recruited 25 hospitalized adults from Dhaka Medical College Hospital between November 2022 and January 2023. The median age of the hospitalized patients was 24.3 years (19.8–49.6), and the gender distribution was 80% male. We collected stool and blood samples from hospitalized individuals at four time points over a 2-month period (day 0, 7, 30, and 60), in the same manner as for the community cohort.

### Prevalence of Kp is high in both community and hospitalized populations

Our previous study found that the average prevalence of Kp among healthy individuals in this community for the 2-month period by PCR was 93% (69.3/75), ranging from 85% in young children (21.2/25) to 99% (25.8/26) in adults (30). In this study, we screened hospitalized adults for Kp using real-time PCR with identical methods. Kp was detected in 100% (25/25) of participants at least once during the 2-month sampling period. Kp was detected at all four time points for 80% (20/25) of these individuals. Continuous detection of Kp at sequential time points was common in both cohorts (i.e., 79% of subjects across 30 days and 69% across 60 days).

### Simultaneous GI carriage of multiple Kp strains is common

Next, we sought to determine whether high Kp carriage prevalence reflected persistent colonization by the same strain or sequential turnover among different strains. We used real-time PCR screening to identify Kp O-antigen types within individual fecal samples. Overall, 90% (90/100) of subjects had at least one time point at which more than one Kp O-type was detected, consistent with co-carriage of multiple strains (data not shown).

To further characterize strain-level carriage, we performed metagenomic sequencing of Kp-enriched fecal cultures utilizing a previously validated method (31, 32). Analyses were restricted to 73 subjects in whom Kp was detected by PCR at all three sampling time points within the first 30 days. Nineteen of the 27 subjects were excluded due to lack of available enriched culture samples for one or more time points. The remaining inclusion group included 16 children under 5 years of age, 15 children aged 5–17 years, 22 healthy adults, and 20 hospitalized adults aged ≥18 years.

**Fig 1:**
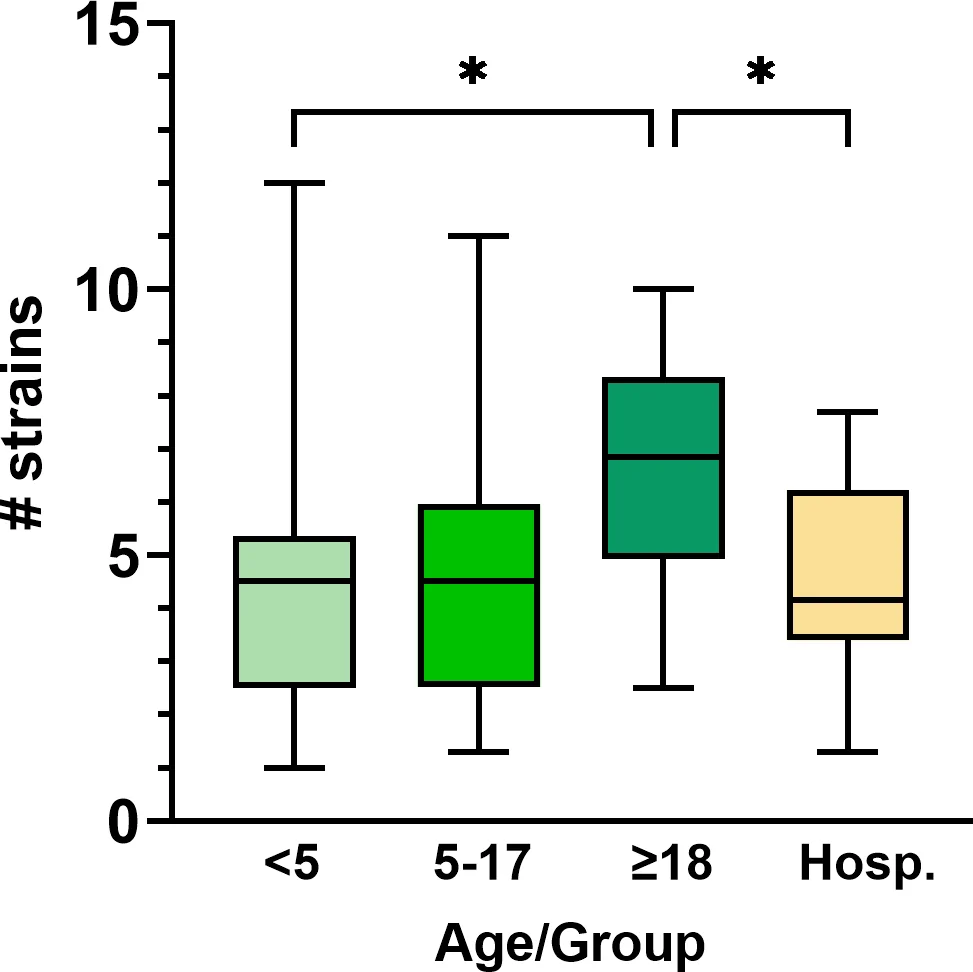
Average number of observed Kp strains per subject over the 30-day screening period (“*” adjusted *P* < 0.05).

We used StrainGE—a tool for deconvolving mixed strain populations in metagenomic data—to profile *Kp* metagenome communities at the strain level. We found that individual subjects often carried multiple Kp strains at the same time (Fig. 1). Over the 30-day period, adults living in the community had a higher mean number of distinct Kp strains per person (*N* = 6.5) than hospitalized individuals (*N* = 4.6, *P* = 0.0226), and children under 5 years old living in the community (*N* = 4.6, *P* = 0.0310; Fig. 1). Several individuals in each group were found to carry 10 or more distinct Kp strains simultaneously.

For comparison, we also performed strain-level analyses for *Escherichia* and *Enterobacter* species detected in the enriched culture samples (Table S1). Multiple *Escherichia* strains were commonly detected within individuals, whereas *Enterobacter* showed lower strain multiplicity (Fig. S1).

### GI carriage of individual strains is transient for most subjects

**Fig 2:**
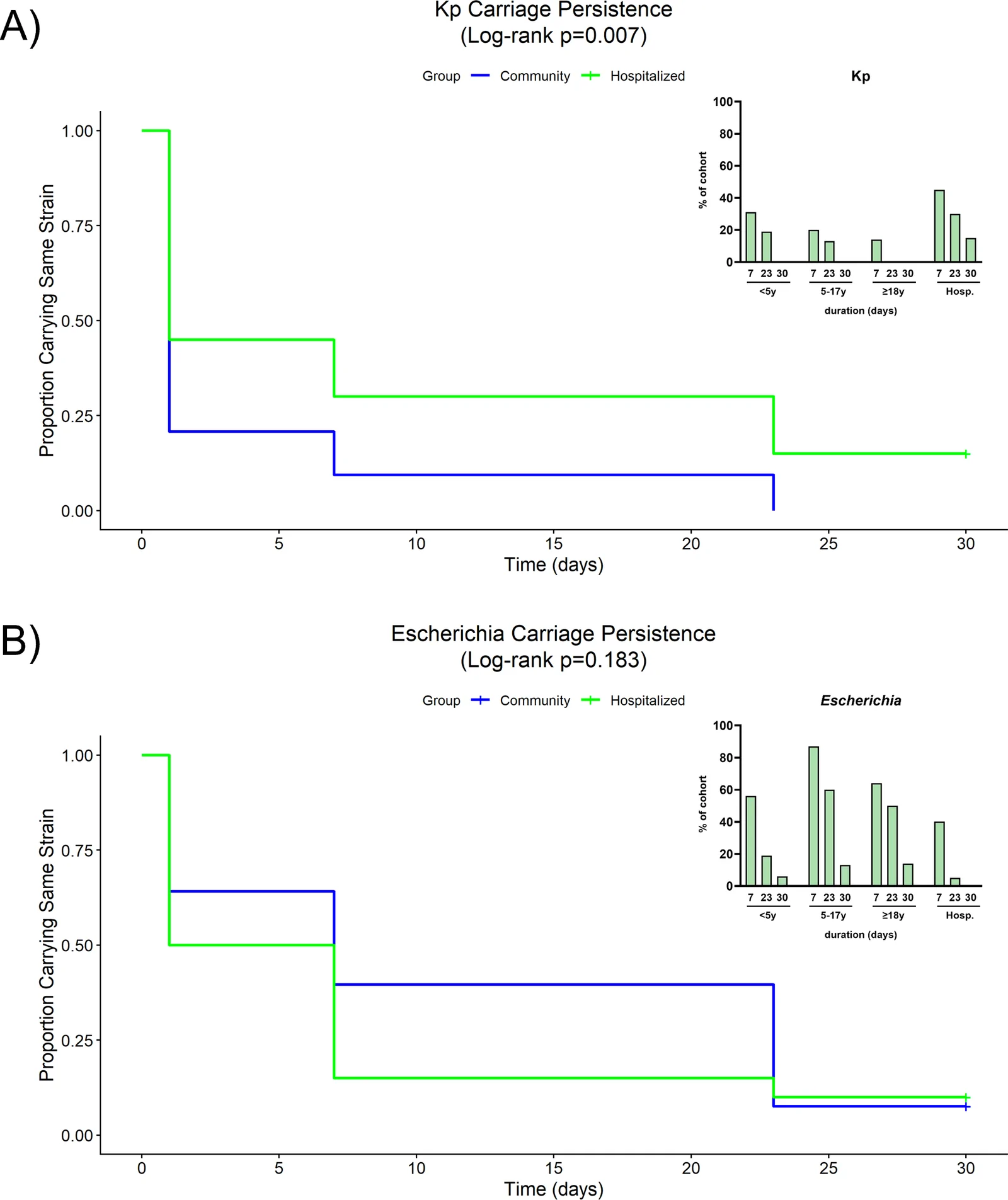
Kaplan–Meier curves for strain carriage duration of (**A**) Kp and (**B**) *Escherichia* spp. by group. Insets show the distribution of subjects with extended GI carriage of one or more strains during the 30-day screening period. Full Cox proportional hazards model results are provided in Table S2.

We used metagenomic data to analyze the persistence of Kp strain carriage in study participants from the community and hospital cohorts. Carriage of individual Kp strains was generally transient in the healthy community across age groups, with sequential detection of a given strain between time points (≥7  days apart) observed in only 21% (11/53) of participants (Fig. 2A). No strains were detected across all three time points within the 30-day sampling period in the community cohort.

In contrast, among hospitalized adults, 45% (9/20) exhibited sequential detection of the same strain for  ≥7  days, and 15% (3/20) maintained at least one strain throughout the 30-day period, including two individuals with persistent carriage of the same strain (KPN234; MLST420). Survival analysis supported these patterns, revealing significantly prolonged carriage among hospitalized adults compared with the community group (log-rank *χ*²  =  7.32, df  =  1, *P*  =  0.0068; HR  =  0.495, 95% CI 0.281–0.871) (Fig. 2A; Table S2).

By comparison, *Escherichia* strain carriage was more stable than Kp across all age groups in the community, with 60.3% (44/73) of participants carrying the same strain for at least one week. The proportion of individuals with sequential detection ranged from 40% (8/20) in hospitalized adults to 87% (13/15) in children aged 5–17 years (Fig. 2B). Consistent with these trends, survival analysis showed no significant differences in *Escherichia* carriage duration between hospital and community cohorts (log-rank *χ*²  = 1.77, df  =  1, *P*  =  0.1834). (Fig. 2B; Table S2).

### High strain diversity

**Fig 3:**
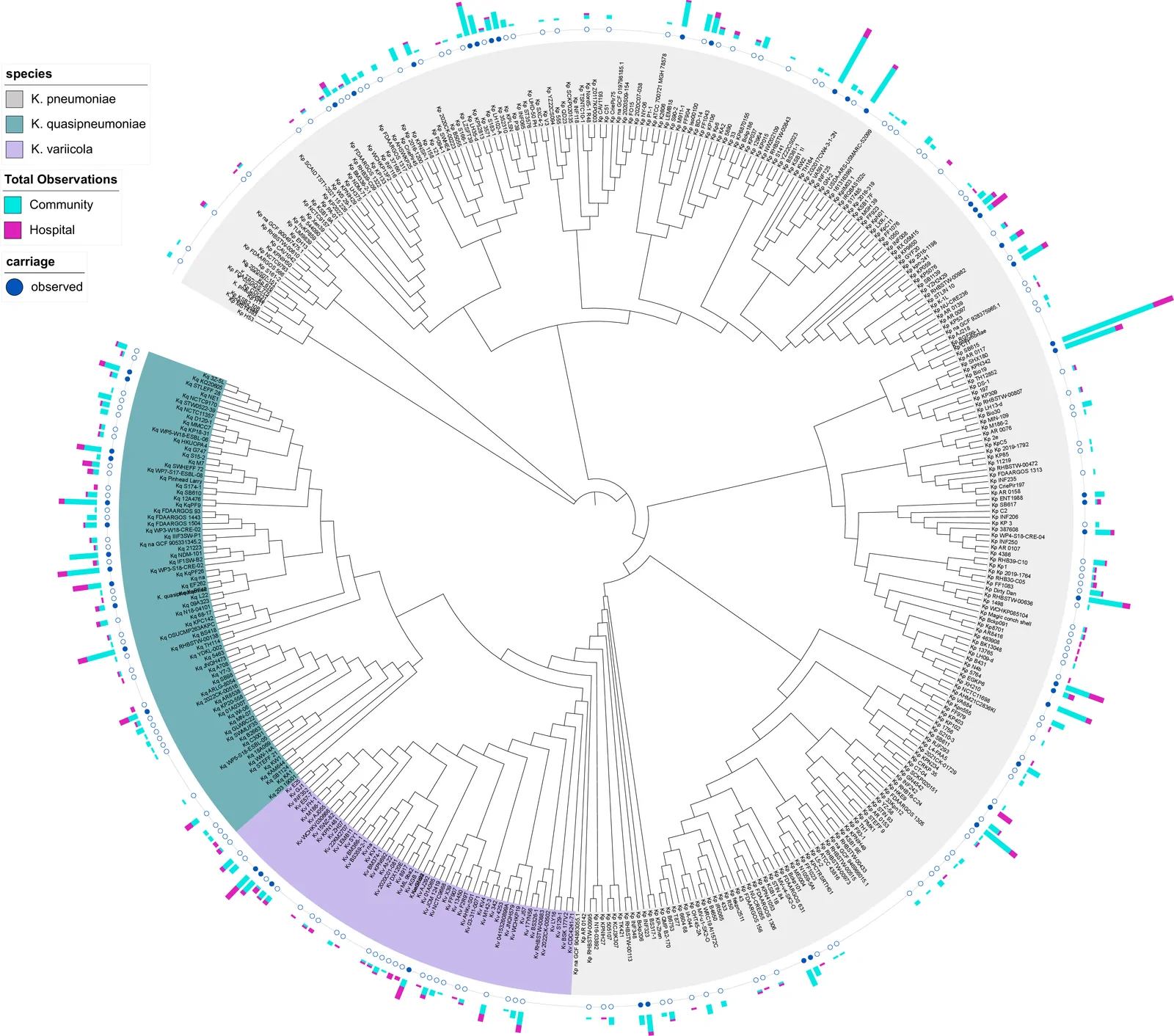
Phylogenetic tree of Kp strains. The 212 observed strains were mapped onto a tree comprised of 405 non-redundant strains from a StrainGST reference database, reduced from >2,000 genomes from NCBI. Branch lengths were minimized for clarity. The outer ring indicates the total number of observations in the cohort. Observations are divided into subjects from the community (light blue) and hospitalized (pink) patients. Strains associated with longer-term carriage, i.e., appearing in the same subject at more than one time point, are indicated by filled blue circles in the inner ring. Detailed data is also provided in Table S6.

Overall, StrainGE identified 212 unique strains within the Kp across the study population. Among the detected Kp strains, the species distribution was 59.6% *K. pneumoniae*, 23.1% *K. quasipneumoniae*, and 11.5% *K. variicola* (Table S3). We compared the total occurrences of each strain between community and hospitalized groups and mapped the phylogenetic relationships to assess potential clustering patterns in strain prevalence and associations with extended carriage events (Fig. 3; Table S6). Phylogenetic mapping revealed broad genetic diversity, spanning multiple clades across Kp phylogroups. Strains commonly overlapped between hospitalized and community populations across age groups, with 62% (131/212) detected in more than one group or population. On a species-level, *K. variicola* was significantly more prevalent in hospitalized subjects (20.8%, 50/240) than in the healthy community (10.5%, 73/697; Fisher’s exact test, *P* = 0.0004). Certain strains also clustered within cohorts: Kpn-241 (MLST37) appeared in 40% (8/20) of hospitalized subjects but only 5.7% (3/53) of healthy subjects (Fisher’s exact test, *P* = 0.004), while KP102 (MLST405) was exclusive to healthy adults (28%, 6/22).

### ESBL and carbapenemase ARGs in community and hospitalized populations

Antibiotic resistance phenotyping performed in our prior study found that among community Kp isolates, 12% were carbapenem-resistant, 8% were ESBL producers, and 9% were multidrug-resistant (30). Although our current study approach does not permit definitive attribution of ARGs to Kp in enriched samples, we analyzed metagenomic data using AMRFinderPlus (33) to identify ARGs for known Kp ESBL (e.g., *bla*SHV, *bla*TEM, *bla*CTX) and carbapenem (e.g., *bla*KPC, *bla*NDM, *bla*VIM, *bla*IMP, *bla*OXA) resistance determinants (34). ESBL ARGs were detected in 96.2% (51/53) of healthy subjects and 85% (17/20) of hospitalized subjects (Table S4). *bla*CTX-M-15 was the most frequently observed ESBL gene and was present in at least one time point for 97.1% (66/68) of ESBL-positive subjects. It was also the only ESBL ARG carried persistently (i.e., detected in all three time points) for 31.8% (21/68) of ESBL-positive subjects (Table 1). Carbapenem-resistance genes were detected in 24.5% (13/53) of healthy subjects and 15% (3/20) of hospitalized subjects (Table 1). No persistent carriage of carbapenem-resistance genes was noted.

**TABLE 1:** Frequency of individuals with an extended-spectrum β-lactamases (ESBL) or carbapenemase (CRB) resistance gene detected for at least one (or all three time points) during the 30-day screening period

	<5 y		5–17 y		≥18 y		Hospitalized		Total	
Timepoints ARG detected	≥1	3	≥1	3	≥1	3	≥1	3	≥1	3
ESBL prevalence (%)	16/16 (100%)	8/16 (50%)	15/15 (100%)	4/15 (27%)	20/22 (91%)	6/22 (27%)	17/20 (85%)	3/20 (15%)	68/73 (93%)	21/73 (29%)
CRB prevalence (%)	4/16 (25%)	0/16 (0%)	4/15 (27%)	0/15 (0%)	5/22 (23%)	0/22 (0%)	3/20 (15%)	0/20 (0%)	16/73 (22%)	0/73 (0%)

### Circulating antibodies to Kp surface polysaccharide antigens increase in an age-dependent manner

**Fig 4:**
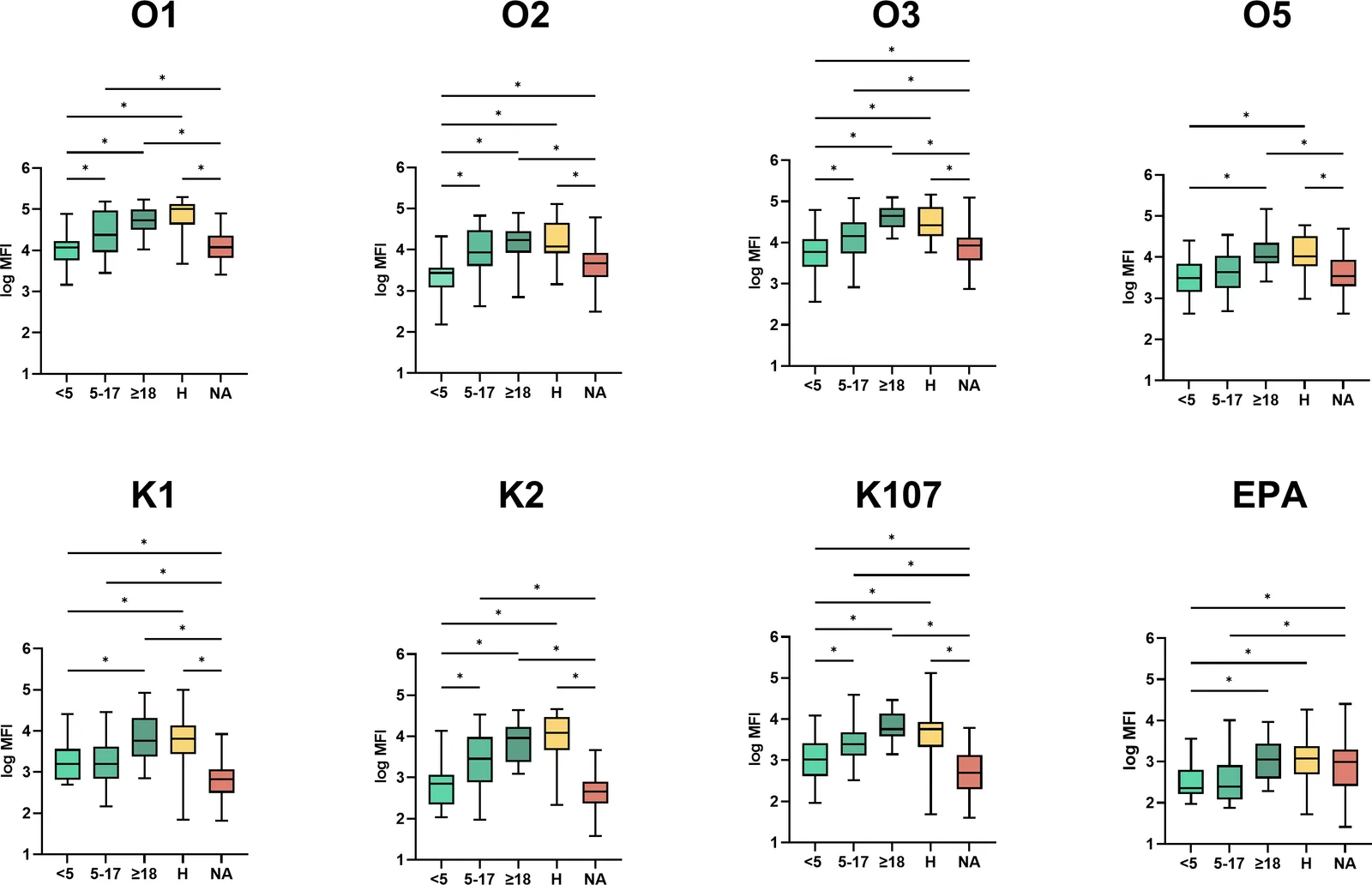
Serum IgG antibody levels to common O and K antigens increase with age. Log-transformed median fluorescent intensity (MFI) values for Kp O- and K-antigens from the baseline (day 0) time point. Age groups are plotted in ascending order (green). Hospitalized adult (“H,” yellow) and North American adult population (“NA,” orange) are included for comparison. Groups sharing an asterisk are significantly different after Benjamini-Krieger-Yekutieli FDR correction (*q* < 0.05); full post hoc results are provided in Table S5. There was no significant difference in response across time points, so plots were limited to day 0 for clarity.

We measured serum antibody responses to selected Kp lipopolysaccharide (O-antigen) and capsule (K-antigen) surface antigens using a custom multiplex bead assay (35). Antibody levels were compared across age groups of healthy subjects and hospitalized individuals (Fig. 4). To compare antibody responses with a cohort with a lower Kp prevalence, we included a group of 36 healthy North American outpatient adults recruited from Massachusetts General Hospital, Boston, MA.

Circulating IgG levels against the evaluated O- and K-antigens were significantly higher in the Bangladeshi cohort compared with North American adults (Fig. 4). Moreover, IgG levels were higher in adults than in young children, with intermediate levels in older children. There were moderate positive correlations between age and antibody levels for all Kp antigens (Pearson *r* values of 0.4473–0.6016, Table S7)

## DISCUSSION

In this study, we defined the strain-level carriage dynamics, antimicrobial resistance gene (ARG) profiles, and circulating anti-Kp antibody responses in community members and hospitalized adults in Dhaka, Bangladesh. Using metagenomic sequencing of Kp-enriched cultures and strain-resolved bioinformatic analyses, we found that while species-level carriage of Kp is nearly continuous in this high-exposure setting, the carriage of individual strains is typically transient. IgG antibody responses to common Kp surface antigens were also common in the study population and increased with subject age, which likely reflects the effect of sustained exposure to Kp that we observed in these subjects.

Kp carriage was highly prevalent across all age groups in the community and was universal among hospitalized adults. Despite this, most Kp strains were detected at only a single time point, with prolonged carriage of the same strain (≥7 days) occurring primarily in young children and hospitalized adults. In contrast, *E. coli* strains, which were also found ubiquitously in our Kp-enrichment cultures, showed substantially greater stability within individuals, a finding consistent with previous reports suggesting *E. coli* strain carriage can persist for weeks to years (36, 37). Together, these findings indicate that, in this community, species-level persistence of Kp reflects frequent acquisition and replacement rather than long-term dominance of individual strains, and that young age and healthcare exposure are associated with longer strain residence times. These observations from a highly endemic population are consistent with prior reports of gut carriage from lower-prevalence settings (16, 29, 38) and collectively highlight the dynamic nature of Kp in the GI tract.

Strain-level analyses revealed extensive genetic diversity within Kp, with frequent simultaneous carriage of multiple strains. Healthy adults carried the highest number of distinct strains, often five to seven over a 30-day period, with some individuals harboring 10 or more. Similar patterns of strain multiplicity were observed for *E. coli*, suggesting high environmental acquisition pressure for multiple *Enterobacterales* species in this setting. The frequency of co-carriage observed here exceeds that reported in most prior studies (25, 29, 31, 39), underscoring the potential for horizontal gene transfer and suggesting that culture-based methods used to identify dominant colonizing strains may not adequately capture the full diversity of carriage.

Antibiotic resistance genes (ARGs), especially ESBLs, were common in both community and hospital settings. The blaCTX-M-15 predominance aligns with other South Asia reports (40, 41) and signals widespread dissemination of this AMR determinant. Carbapenem-resistance genes were less common but present in 22% of subjects, indicating potential community reservoirs of high-priority ARGs. Hospitalized adults showed greater ESBL gene diversity, likely due to antibiotic exposure or hospital-related selection.

Previous studies have indicated that host immune status is a key determinant of Kp gastrointestinal colonization and clearance (42–46), consistent with the rapid strain turnover observed in most individuals in this cohort. Bangladeshi participants exhibited substantially higher IgG levels to Kp O and K antigens than North American adults, with concentrations increasing with age. At a population level, the association between high levels of exposure and elevated anti-Kp antibody levels suggests that serologic data could serve as a valuable tool for estimating community Kp burden and identifying dominant antigens in circulation.

This study has several limitations. First, StrainGST derives signatures from unique genomic regions (k-mers) for each strain in the reference and sample databases, so strain assignments reflect the closest reference match rather than the strain’s complete genome sequence. Second, the healthy cohort for this study came from a single urban neighborhood and followed for a limited period, and strain-level metagenomic analyses were restricted to a subset of participants and time points. Third, serological analyses of O and K antigen reactivity did not capture the full diversity of observed Kp antigen types, although the included O-antigens are estimated to represent 80–90% of circulating strains (47–49). Fourth, participants in the community cohort were not systematically recruited at the household level, and ward-level metadata for hospitalized patients were insufficient to assess clustering. Finally, although enrichment-based metagenomics is substantially more sensitive than direct stool sequencing, low-abundance strains may have been missed, potentially leading to underestimation of carriage duration.

In summary, we demonstrate that gastrointestinal carriage of *K pneumoniae* in Dhaka, Bangladesh, is characterized by extensive strain diversity, frequent co-carriage, a substantial burden of antimicrobial resistance genes, and rapid turnover of individual strains. The data further suggest that sustained environmental exposure may drive adaptive immune responses, as evidenced by significantly higher circulating IgG levels against key Kp surface antigens than in North American controls. These findings have important implications for understanding colonization dynamics, transmission, and the development of preventive strategies.

## MATERIALS AND METHODS

### Subjects and sample collection

Healthy subjects were recruited in the Mirpur neighborhood of Dhaka, Bangladesh, and hospitalized subjects were recruited from Dhaka Medical College Hospital between August 2022 and January 2023 under BMRC protocol #43617082021 and MGH IRB protocol #2021P002799 (refer to Table S8 for demographic and clinical details). Stool samples were collected at four time points over a 60-day period (day 0, 7, 30, and 60) in sterile containers without transport media and transported under a maintained cold chain to a central laboratory for processing on the day of collection, as previously described (30). Contemporaneous venous blood samples were collected in serum tubes, transported to a central laboratory for processing, and stored at −80°C until transfer to MGH for analysis. Reported values for 2-month Kp prevalence in the community cohort were the average of Kp-positive individuals at each of the four time points. North American healthy adult blood samples were collected as baseline controls in an international traveler study and were collected in Boston, MA, from August 2019 to March 2020 under MGH IRB protocol #2019P001392. Samples were stored at −80°C until analysis.

### Bacterial culture

Kp-enrichment cultures were performed as described previously (30). Briefly, sterile-filtered phosphate-buffered saline with 15% glycerol (vol/vol), pH 7.4 (1× PBSG) was added to stool at a concentration of 200 mg/mL, and the mixture was homogenized by vortexing. The homogenate was plated on Simmons Citrate Agar (HiMedia Laboratories, India) supplemented with 1% inositol (Carl Roth, USA) (SCAI) and incubated at 37°C for up to 48 h. Kp-enriched plate-swipe samples were prepared by dragging a sterile swab across the entire agar plate and resuspending in sterile 1× PBSG. Stocks were stored at −80°C until testing.

### Real-time PCR and metagenomic sequencing

DNA was extracted from 0.2 mL of the plate-swipe sample using the MagMax Microbiome Ultra Kit on a KingFisher Flex instrument (Thermo Fisher Scientific, Waltham, MA, USA). Extracts were screened by real-time PCR to confirm the presence of Kp and establish O-antigen type(s) present using established methods (50, 51). Sequencing libraries were prepared at the Vanderbilt University Medical Center sequencing core (VANTAGE, Nashville, TN, USA) using the Twist Library Prep Kit (Twist Bioscience, San Francisco, CA, USA). Libraries were sequenced on an Illumina NovaSeq XP (San Diego, CA, USA) with 150 bp paired-end reads. Average yields were 10 million reads per sample for metagenomic sequencing, providing ≥250× coverage.

### Metagenomic analyses

Reads were trimmed for quality and adapter content using Trim Galore (v0.6.5), assessed with FastQC (v0.11.8), and screened for human reads using KneadData (v0.7.2), after which human reads were removed prior to downstream analyses. Metagenomic taxonomic profiles were generated using MetaPhlAn (v4.0). Strain-level assignments were performed with the StrainGE toolkit (v1.3.9). The StrainGE Genome Sequence Tool (StrainGST) reference database was compiled from >2,000 complete Kp genomes downloaded from NCBI GenBank in November 2023. Reference databases for *Escherichia* and *Enterobacter* were also included for comparison and to improve assignment specificity. Antimicrobial resistance gene detection was performed using AMRFinderPlus (v4.0.3). Pan-genome clustering and phylogenetic analyses were conducted using Panaroo (v1.5.1), and the resulting tree was visualized and annotated in iTOL (v7.2.1).

### Antibody profiling

Circulating IgG antibody levels against Kp surface antigens (i.e., O1, O2, O3, O5, K1, K2, K107) were quantified using a custom multiplexed magnetic bead–based assay developed by our laboratory, as previously described (37). Briefly, serum samples were diluted 1:100 in assay buffer (1:1,000 final dilution) and incubated with beads coated with purified Kp O- and K-polysaccharides or their carrier protein (*Pseudomonas aeruginosa* exotoxin A, EPA). Bound antibodies were detected using PE-labeled, human IgG-specific secondary antibody (Southern Biotechnology, Birmingham, AL, USA) diluted 1:150 in assay buffer. Background-subtracted median fluorescence intensity (MFI) values were calculated from ≥60 beads per antigen acquired on a Luminex FlexMap 3D instrument (Austin, TX, USA).

### Statistical analysis

One-way ANOVA (with Benjamini, Krieger, and Yekutieli post hoc test), Fisher’s exact test, and Pearson correlation analyses were conducted in GraphPad Prism (v10.4.0) to evaluate differences in Kp strain carriage, species prevalence, and antibody levels across age groups and populations.

Kp and *Escherichia* carriage survival analyses were conducted separately using Kaplan–Meier curves, log-rank tests, and Cox proportional hazards models across the two groups (total community vs hospitalized). Time to loss of carriage was defined as the number of days from initial strain detection to first documented clearance, with subjects censored at their last collection if the strain persisted. Participants without evidence of continuous carriage between collections were assigned a carriage duration of one day. All analyses used the survival and survminer packages in R (v4.5.3), with two-sided *P*  <  0.05 considered statistically significant.

## Data Availability

Sequencing data have been deposited to the NCBI Sequence Read Archive under BioProject accession PRJNA1374131. Individual sample accession numbers are listed in Table S9. All metadata associated with BioSamples are publicly accessible via NCBI.

## SUPPLEMENTAL MATERIAL

Supplemental figure and tables spectrum.01651-26-s0001.docx Fig. S1 and Tables S2, S5 to S7.

Table S1 spectrum.01651-26-s0002.xlsx metaphlan data.

Table S3 spectrum.01651-26-s0003.xlsx StrainGST data.

Table S4 spectrum.01651-26-s0004.xlsb AMRFinder data.

Table S8 spectrum.01651-26-s0005.xlsx Demographic and clinical metadata.

Table S9 spectrum.01651-26-s0006.xlsx NCBI Biosample accession numbers.

ASM does not own the copyrights to Supplemental Material that may be linked to, or accessed through, an article. The authors have granted ASM a non-exclusive, world-wide license to publish the Supplemental Material files. Please contact the corresponding author directly for reuse.
