# Supplementary material for: High exposure, rapid turnover: strain-level dynamics of Klebsiella pneumoniae carriage in community and hospitalized populations in Bangladesh
Source: Microbiol Spectr. 2026 Aug 25;14(10):e01651-26. doi: 10.1128/spectrum.01651-26 (PMC13689179; doi:10.1128/spectrum.01651-26)
Supplement: Supplementary file 1 [file spectrum.01651-26-s0001.docx]

**Supplemental Tables and Figures**

**Table S1**: Metaphlan data (excel table)

**Figure S1.** (A) Total and (B) average number of observed strains across 3 timepoints.

**Table S2.** Kaplan–Meier survival log-rank tests, and Cox proportional-hazards model data

| **Kp Carriage** |  |  | |  | |  | |  |  |  |  |  |
| --- | --- | --- | --- | --- | --- | --- | --- | --- | --- | --- | --- | --- |
| **Log-rank test** | χ2 =7.32, df=1, p=0.0068 |  | |  | |  | |  |  |  |  |  |
| **Cox Proportion Hazard Analysis** | | |  | |  | |  |  |  |  |  |  |
| **Term** | **coef (log HR)** | **exp(coef) (HR)** | | **se(coef)** | | **z** | | **p** | **exp(coef)** | **exp(-coef)** | **lower .95** | **upper .95** |
| Group Hospitalized | -0.703 | 0.495 | | 0.288 | | -2.439 | | 0.015 | 0.495 | 2.020 | 0.281 | 0.871 |
|  |  |  | |  | |  | |  |  |  |  |  |
| **Model Test** | **value** | **df** | | **p** | |  | |  |  |  |  |  |
| Likelihood ratio test | 6.58 | 1 | | 0.01 | |  | |  |  |  |  |  |
| Wald test | 5.95 | 1 | | 0.01 | |  | |  |  |  |  |  |
| Score (log‑rank) test | 6.15 | 1 | | 0.01 | |  | |  |  |  |  |  |
|  |  |  | |  | |  | |  |  |  |  |  |
| **Concordance** | 0.624 | **se** | | 0.057 | |  | |  |  |  |  |  |
|  |  |  | |  | |  | |  |  |  |  |  |
| **Escherichia Carriage** |  |  | |  | |  | |  |  |  |  |  |
| **Log-rank test** | χ2=1.77, df=1, p=0.1834 |  | |  | |  | |  |  |  |  |  |
| **Cox Proportion Hazard Analysis** | | |  | |  | |  |  |  |  |  |  |
| **Term** | **coef (log HR)** | **exp(coef) (HR)** | | **se(coef)** | | **z** | | **p** | **exp(coef)** | **exp(-coef)** | **lower .95** | **upper .95** |
| Group Hospitalized | 0.299 | 1.349 | | 0.278 | | 1.076 | | 0.282 | 1.349 | 0.742 | 0.782 | 2.324 |
|  |  |  | |  | |  | |  |  |  |  |  |
| **Model Test** | **value** | **df** | | **p** | |  | |  |  |  |  |  |
| Likelihood ratio test | 1.11 | 1 | | 0.3 | |  | |  |  |  |  |  |
| Wald test | 1.16 | 1 | | 0.3 | |  | |  |  |  |  |  |
| Score (log‑rank) test | 1.17 | 1 | | 0.3 | |  | |  |  |  |  |  |
|  |  |  | |  | |  | |  |  |  |  |  |
| **Concordance** | 0.561 | **se** | | 0.04 | |  | |  |  |  |  |  |

**Table S3.** StrainGST output and breakdown of observed Klebsiella, Escherichia and Enterobacter species (excel table)

**Table S4.** AMRFinderPlus output (excel table)

**Table S5. Serological analysis – One-way ANOVA summarized results**

| **ANOVA Summary** | **F (4,131)** | **p** | **Benjamini, Krieger**  **and Yekutieli test** | **Mean**  **diff.** | **SE of**  **diff.** | **q value** | **Individual**  **P Value** |
| --- | --- | --- | --- | --- | --- | --- | --- |
| O1 IgG | 18.59 | <0.0001 | <5  vs. 5-17 | -0.3760 | 0.1232 | 0.0010 | 0.0028 |
|  |  |  | <5  vs. 18+ | -0.7204 | 0.1208 | <0.0001 | <0.0001 |
|  |  |  | <5  vs. H | -0.8041 | 0.1220 | <0.0001 | <0.0001 |
|  |  |  | <5  vs. NA | -0.1056 | 0.1123 | 0.0813 | 0.3485 |
|  |  |  | 5-17 vs. 18+ | -0.3444 | 0.1221 | 0.0017 | 0.0055 |
|  |  |  | 5-17 vs. H | -0.4281 | 0.1232 | 0.0003 | 0.0007 |
|  |  |  | 5-17 vs. NA | 0.2704 | 0.1136 | 0.0049 | 0.0188 |
|  |  |  | 18+ vs. H | -0.0837 | 0.1208 | 0.1029 | 0.4898 |
|  |  |  | 18+ vs. NA | 0.6148 | 0.1110 | <0.0001 | <0.0001 |
|  |  |  | H vs. NA | 0.6985 | 0.1123 | <0.0001 | <0.0001 |
| O2 IgG | 13.69 | <0.0001 | <5  vs. 5-17 | -0.6011 | 0.1462 | <0.0001 | <0.0001 |
|  |  |  | <5  vs. 18+ | -0.8238 | 0.1433 | <0.0001 | <0.0001 |
|  |  |  | <5  vs. H | -0.8534 | 0.1447 | <0.0001 | <0.0001 |
|  |  |  | <5  vs. NA | -0.2643 | 0.1332 | 0.0295 | 0.0492 |
|  |  |  | 5-17 vs. 18+ | -0.2227 | 0.1448 | 0.0590 | 0.1264 |
|  |  |  | 5-17 vs. H | -0.2523 | 0.1462 | 0.0455 | 0.0867 |
|  |  |  | 5-17 vs. NA | 0.3368 | 0.1348 | 0.0096 | 0.0137 |
|  |  |  | 18+ vs. H | -0.0296 | 0.1433 | 0.3514 | 0.8368 |
|  |  |  | 18+ vs. NA | 0.5595 | 0.1317 | <0.0001 | <0.0001 |
|  |  |  | H vs. NA | 0.5891 | 0.1332 | <0.0001 | <0.0001 |
| O3 IgG | 17.74 | <0.0001 | <5  vs. 5-17 | -0.3919 | 0.1337 | 0.0019 | 0.0040 |
|  |  |  | <5  vs. 18+ | -0.9040 | 0.1311 | <0.0001 | <0.0001 |
|  |  |  | <5  vs. H | -0.7812 | 0.1323 | <0.0001 | <0.0001 |
|  |  |  | <5  vs. NA | -0.1969 | 0.1218 | 0.0407 | 0.1084 |
|  |  |  | 5-17 vs. 18+ | -0.5121 | 0.1324 | 0.0001 | 0.0002 |
|  |  |  | 5-17 vs. H | -0.3893 | 0.1337 | 0.0019 | 0.0042 |
|  |  |  | 5-17 vs. NA | 0.1950 | 0.1233 | 0.0407 | 0.1162 |
|  |  |  | 18+ vs. H | 0.1228 | 0.1311 | 0.1104 | 0.3504 |
|  |  |  | 18+ vs. NA | 0.7071 | 0.1204 | <0.0001 | <0.0001 |
|  |  |  | H vs. NA | 0.5843 | 0.1218 | <0.0001 | <0.0001 |
| O5 IgG | 9.45 | <0.0001 | <5  vs. 5-17 | -0.1339 | 0.1374 | 0.1991 | 0.3318 |
|  |  |  | <5  vs. 18+ | -0.6260 | 0.1347 | <0.0001 | <0.0001 |
|  |  |  | <5  vs. H | -0.5567 | 0.1360 | 0.0001 | <0.0001 |
|  |  |  | <5  vs. NA | -0.0931 | 0.1252 | 0.2406 | 0.4583 |
|  |  |  | 5-17 vs. 18+ | -0.4921 | 0.1361 | 0.0004 | 0.0004 |
|  |  |  | 5-17 vs. H | -0.4228 | 0.1374 | 0.0018 | 0.0026 |
|  |  |  | 5-17 vs. NA | 0.0407 | 0.1267 | 0.3143 | 0.7484 |
|  |  |  | 18+ vs. H | 0.0693 | 0.1347 | 0.2837 | 0.6080 |
|  |  |  | 18+ vs. NA | 0.5328 | 0.1238 | <0.0001 | <0.0001 |
|  |  |  | H vs. NA | 0.4636 | 0.1252 | 0.0003 | 0.0003 |
| K1 IgG | 17.21 | <0.0001 | <5  vs. 5-17 | -0.0148 | 0.1578 | 0.1943 | 0.9254 |
|  |  |  | <5  vs. 18+ | -0.6154 | 0.1547 | <0.0001 | 0.0001 |
|  |  |  | <5  vs. H | -0.4948 | 0.1562 | 0.0008 | 0.0019 |
|  |  |  | <5  vs. NA | 0.4206 | 0.1437 | 0.0011 | 0.0040 |
|  |  |  | 5-17 vs. 18+ | -0.6006 | 0.1563 | <0.0001 | 0.0002 |
|  |  |  | 5-17 vs. H | -0.4799 | 0.1578 | 0.0010 | 0.0028 |
|  |  |  | 5-17 vs. NA | 0.4354 | 0.1455 | 0.0010 | 0.0033 |
|  |  |  | 18+ vs. H | 0.1207 | 0.1547 | 0.1019 | 0.4366 |
|  |  |  | 18+ vs. NA | 1.0360 | 0.1421 | <0.0001 | <0.0001 |
|  |  |  | H vs. NA | 0.9154 | 0.1437 | <0.0001 | <0.0001 |
| K2 IgG | 36.75 | <0.0001 | <5  vs. 5-17 | -0.6066 | 0.1516 | <0.0001 | 0.0001 |
|  |  |  | <5  vs. 18+ | -1.0350 | 0.1486 | <0.0001 | <0.0001 |
|  |  |  | <5  vs. H | -1.1810 | 0.1500 | <0.0001 | <0.0001 |
|  |  |  | <5  vs. NA | 0.1645 | 0.1381 | 0.0550 | 0.2358 |
|  |  |  | 5-17 vs. 18+ | -0.4282 | 0.1502 | 0.0013 | 0.0051 |
|  |  |  | 5-17 vs. H | -0.5739 | 0.1516 | <0.0001 | 0.0002 |
|  |  |  | 5-17 vs. NA | 0.7711 | 0.1398 | <0.0001 | <0.0001 |
|  |  |  | 18+ vs. H | -0.1457 | 0.1486 | 0.0690 | 0.3285 |
|  |  |  | 18+ vs. NA | 1.1990 | 0.1365 | <0.0001 | <0.0001 |
|  |  |  | H vs. NA | 1.3450 | 0.1381 | <0.0001 | <0.0001 |
| K107 IgG | 18.15 | <0.0001 | <5  vs. 5-17 | -0.3998 | 0.1588 | 0.0059 | 0.0130 |
|  |  |  | <5  vs. 18+ | -0.8152 | 0.1557 | <0.0001 | <0.0001 |
|  |  |  | <5  vs. H | -0.5779 | 0.1572 | 0.0002 | 0.0003 |
|  |  |  | <5  vs. NA | 0.2599 | 0.1447 | 0.0295 | 0.0748 |
|  |  |  | 5-17 vs. 18+ | -0.4154 | 0.1573 | 0.0049 | 0.0093 |
|  |  |  | 5-17 vs. H | -0.1781 | 0.1588 | 0.0832 | 0.2642 |
|  |  |  | 5-17 vs. NA | 0.6597 | 0.1465 | <0.0001 | <0.0001 |
|  |  |  | 18+ vs. H | 0.2372 | 0.1557 | 0.0455 | 0.1300 |
|  |  |  | 18+ vs. NA | 1.0750 | 0.1431 | <0.0001 | <0.0001 |
|  |  |  | H vs. NA | 0.8378 | 0.1447 | <0.0001 | <0.0001 |
| EPA IgG | 5.178 | 0.0007 | <5  vs. 5-17 | -0.0516 | 0.1636 | 0.4393 | 0.7531 |
|  |  |  | <5  vs. 18+ | -0.5315 | 0.1604 | 0.0031 | 0.0012 |
|  |  |  | <5  vs. H | -0.5422 | 0.1620 | 0.0031 | 0.0011 |
|  |  |  | <5  vs. NA | -0.3773 | 0.1491 | 0.0132 | 0.0126 |
|  |  |  | 5-17 vs. 18+ | -0.4799 | 0.1621 | 0.0048 | 0.0036 |
|  |  |  | 5-17 vs. H | -0.4906 | 0.1636 | 0.0048 | 0.0033 |
|  |  |  | 5-17 vs. NA | -0.3257 | 0.1509 | 0.0286 | 0.0327 |
|  |  |  | 18+ vs. H | -0.0107 | 0.1604 | 0.4971 | 0.9468 |
|  |  |  | 18+ vs. NA | 0.1542 | 0.1474 | 0.1952 | 0.2975 |
|  |  |  | H vs. NA | 0.1649 | 0.1491 | 0.1952 | 0.2707 |

**Table S6. Distribution of observed Kp strains**

| **Species** | **Strain** | **Genebank Accession** | **MLST** | **Total** | **<5y** | **5-17y** | **≥18y** | **Hospital** | **Subgroup restricted*** | **Carriage**** |
| --- | --- | --- | --- | --- | --- | --- | --- | --- | --- | --- |
| *K. pneumoniae* | Bio19 | CP096810.1 | 530 | 43 | 7 | 8 | 18 | 10 | No | Yes |
| *K. pneumoniae* | TH12852 | CP087122.1 | 2643 | 25 | 4 | 3 | 14 | 4 | No | Yes |
| *K. pneumoniae* | KSB1 1I | CP031789.1 | 661 | 24 | 8 | 3 | 10 | 3 | No | Yes |
| *K. pneumoniae* | INF125 | CP031795.1 | 45 | 19 | 5 | 4 | 8 | 2 | No | Yes |
| *K. pneumoniae* | FF1083 | CP077812.1 | 5036 | 19 | 4 | 1 | 10 | 4 | No | Yes |
| *K. pneumoniae* | SCKP020151 | CP043585.1 | 35 | 18 | 4 | 0 | 10 | 4 | No | Yes |
| *K. pneumoniae* | 5764 | CP132713.1 | 17 | 17 | 3 | 6 | 5 | 3 | No | Yes |
| *K. pneumoniae* | LH09-d | CP100542.1 | 20 | 16 | 3 | 3 | 5 | 5 | No | Yes |
| *K. pneumoniae* | S183-1 | CP063927.1 | 2158 | 15 | 2 | 5 | 6 | 2 | No | Yes |
| *K. pneumoniae* | NU-CRE236 | CP061380.1 | 37 | 14 | 4 | 2 | 5 | 3 | No | Yes |
| *K. pneumoniae* | LEMB18 | CP045782.1 | 629 | 11 | 3 | 3 | 4 | 1 | No | Yes |
| *K. pneumoniae* | kpn-241 | CP053666.1 | 37 | 11 | 0 | 1 | 2 | 8 | No | Yes |
| *K. pneumoniae* | Bckp206 | CP050845.1 | 228 | 10 | 0 | 3 | 5 | 2 | No | Yes |
| *K. pneumoniae* | Iso00100 | CP095145.1 | 152 | 9 | 2 | 2 | 3 | 2 | No | Yes |
| *K. pneumoniae* | LH102-A | CP035194.1 | 87 | 9 | 3 | 4 | 2 | 0 | No | Yes |
| *K. pneumoniae* | FF904 | CP077810.1 | 105 | 9 | 0 | 0 | 5 | 4 | No | No |
| *K. pneumoniae* | Kp2016-1198 | WPBA00000000.1 | 2167 | 8 | 1 | 2 | 4 | 1 | No | Yes |
| *K. pneumoniae* | KSB1 1B | LR890291.1 | 1213 | 8 | 3 | 3 | 2 | 0 | No | Yes |
| *K. pneumoniae* | P39 | CP040351.1 | 412 | 8 | 1 | 2 | 5 | 0 | No | Yes |
| *K. pneumoniae* | 387608 | CP132670.1 | 29 | 7 | 3 | 1 | 2 | 1 | No | Yes |
| *K. pneumoniae* | RHBSTW-00982 | CP056216.1 | 37 | 7 | 0 | 2 | 2 | 3 | No | Yes |
| *K. pneumoniae* | 121 | CP031849.1 | 2570 | 7 | 2 | 2 | 3 | 0 | No | No |
| *K. pneumoniae* | 4386 | CP103605.1 | 29 | 7 | 1 | 1 | 4 | 1 | No | No |
| *K. pneumoniae* | CT-04 | CP081891.1 | 1117 | 7 | 2 | 0 | 4 | 1 | No | No |
| *K. pneumoniae* | RHB30-C05 | CP057313.1 | 966 | 7 | 2 | 0 | 4 | 1 | No | No |
| *K. pneumoniae* | XH210 | CP052761.1 | 17 | 7 | 2 | 1 | 4 | 0 | No | No |
| *K. pneumoniae* | KP133 | JAPMWS000000000.1 | 23 | 6 | 0 | 2 | 1 | 3 | No | Yes |
| *K. pneumoniae* | KP102 | JAHMON000000000.1 | 43 | 6 | 0 | 0 | 6 | 0 | Yes | Yes |
| *K. pneumoniae* | KPLSN | CP132990.1 | 412 | 6 | 0 | 1 | 4 | 1 | No | Yes |
| *K. pneumoniae* | 33 | NGXS00000000.1 | 1519 | 6 | 1 | 2 | 3 | 0 | No | No |
| *K. pneumoniae* | SWHE4 | CP055057.1 | 2601 | 5 | 1 | 2 | 1 | 1 | No | Yes |
| *K. pneumoniae* | 371661 | CP132673.1 | 3586 | 5 | 2 | 1 | 2 | 0 | No | Yes |
| *K. pneumoniae* | B431 | CP110661.1 | 20 | 5 | 2 | 0 | 0 | 3 | No | Yes |
| *K. pneumoniae* | IA-044 | CP081889.1 | 107 | 5 | 2 | 0 | 3 | 0 | No | No |
| *K. pneumoniae* | K-1L | CP072460.1 | 727 | 5 | 2 | 0 | 3 | 0 | No | No |
| *K. pneumoniae* | KP-Zhen | CP053862.1 | 1660 | 5 | 0 | 1 | 3 | 1 | No | No |
| *K. pneumoniae* | M186-2 | CP063930.1 | 111 | 5 | 2 | 3 | 0 | 0 | No | No |
| *K. pneumoniae* | INF348 | LR890323.1 | 34 | 4 | 2 | 1 | 1 | 0 | No | Yes |
| *K. pneumoniae* | STIN 84 | CP054997.1 | 315 | 4 | 2 | 0 | 2 | 0 | No | Yes |
| *K. pneumoniae* | 505107 | CP132603.1 | 592 | 4 | 1 | 2 | 1 | 0 | No | No |
| *K. pneumoniae* | BD-14 | CP082975.1 | 152 | 4 | 0 | 1 | 2 | 1 | No | No |
| *K. pneumoniae* | INF065 | LR890665.1 | 2998 | 4 | 1 | 2 | 1 | 0 | No | No |
| *K. pneumoniae* | KP106 | CP089874.1 | 76 | 4 | 0 | 0 | 2 | 2 | No | No |
| *K. pneumoniae* | MGH 39 | CP072949.1 | 1583 | 4 | 1 | 2 | 1 | 0 | No | No |
| *K. pneumoniae* | KPN234 | CP072653.1 | 420 | 3 | 0 | 0 | 0 | 3 | Yes | Yes |
| *K. pneumoniae* | ENT1988 | CP061350.1 | 86 | 3 | 1 | 0 | 2 | 0 | No | Yes |
| *K. pneumoniae* | GYF20 | CP136254.1 | 896 | 3 | 0 | 0 | 3 | 0 | Yes | Yes |
| *K. pneumoniae* | 5890 | CP132705.1 | 76 | 3 | 1 | 0 | 1 | 1 | No | No |
| *K. pneumoniae* | AHSWKP25 | CP091048.1 | 464 | 3 | 1 | 1 | 1 | 0 | No | No |
| *K. pneumoniae* | AR 0153 | CP028932.1 | 14 | 3 | 0 | 2 | 1 | 0 | No | No |
| *K. pneumoniae* | Kp2016-1200 | WPAZ00000000.1 | 25 | 3 | 1 | 1 | 1 | 0 | No | No |
| *K. pneumoniae* | Kp2019-1764 | CP085099.1 | 292 | 3 | 0 | 1 | 0 | 2 | No | No |
| *K. pneumoniae* | MV-u1-SK2-O | CP085866.1 | 219 | 3 | 0 | 0 | 3 | 0 | Yes | No |
| *K. pneumoniae* | NCTC9793 | LR134280.1 | 252 | 3 | 0 | 0 | 1 | 2 | No | No |
| *K. pneumoniae* | Nord5-1 R48 | CP091594.1 | 147 | 3 | 1 | 1 | 0 | 1 | No | No |
| *K. pneumoniae* | SB1139 | CP084859.1 | 37 | 3 | 0 | 0 | 2 | 1 | No | No |
| *K. pneumoniae* | SB617 | CP084825.1 | 55 | 2 | 0 | 0 | 1 | 1 | No | Yes |
| *K. pneumoniae* | TH164 | CP035210.1 | 873 | 2 | 1 | 0 | 1 | 0 | No | Yes |
| *K. pneumoniae* | 352310 | CP132609.1 | 3663 | 2 | 0 | 1 | 0 | 1 | No | No |
| *K. pneumoniae* | 3571 | CP103614.1 | 3923 | 2 | 0 | 0 | 2 | 0 | Yes | No |
| *K. pneumoniae* | Bio30 | CP093856.1 | 2943 | 2 | 0 | 0 | 1 | 1 | No | No |
| *K. pneumoniae* | BS317-1 | CP063936.1 | 1035 | 2 | 2 | 0 | 0 | 0 | Yes | No |
| *K. pneumoniae* | C51 | CP042481.1 | 12 | 2 | 2 | 0 | 0 | 0 | Yes | No |
| *K. pneumoniae* | F93-1 | CP026160.1 | 343 | 2 | 1 | 0 | 1 | 0 | No | No |
| *K. pneumoniae* | FDAARGOS 1305 | CP069847.1 | 4 | 2 | 0 | 1 | 1 | 0 | No | No |
| *K. pneumoniae* | FDAARGOS 1317 | CP069961.1 | 36 | 2 | 1 | 0 | 1 | 0 | No | No |
| *K. pneumoniae* | FDAARGOS 1322 | CP070037.1 | 298 | 2 | 0 | 0 | 1 | 1 | No | No |
| *K. pneumoniae* | FDAARGOS 156 | CP014123.1 | 13 | 2 | 0 | 0 | 1 | 1 | No | No |
| *K. pneumoniae* | FF923 | CP077809.1 | 5034 | 2 | 1 | 1 | 0 | 0 | No | No |
| *K. pneumoniae* | hvKP859 | CP101790.1 | 218 | 2 | 0 | 1 | 0 | 1 | No | No |
| *K. pneumoniae* | INF116 | CP031792.1 | 36 | 2 | 0 | 0 | 1 | 1 | No | No |
| *K. pneumoniae* | INF242 | LR890624.1 | 3072 | 2 | 0 | 1 | 0 | 1 | No | No |
| *K. pneumoniae* | IRQBAS102c | CP071150.1 | 1731 | 2 | 1 | 1 | 0 | 0 | No | No |
| *K. pneumoniae* | Kp1 | JAPZGB000000000.1 | 490 | 2 | 0 | 1 | 0 | 1 | No | No |
| *K. pneumoniae* | Kp3 | JADDLH000000000.1 | 15 | 2 | 0 | 1 | 1 | 0 | No | No |
| *K. pneumoniae* | KP309 | CP089880.1 | 432 | 2 | 1 | 0 | 0 | 1 | No | No |
| *K. pneumoniae* | KpC11 | CP065831.1 | 225 | 2 | 0 | 0 | 0 | 2 | Yes | No |
| *K. pneumoniae* | KSB2 10B | LR890484.1 | 323 | 2 | 0 | 1 | 1 | 0 | No | No |
| *K. pneumoniae* | Magic conch shell | CP065457.1 | 7701 | 2 | 0 | 0 | 1 | 1 | No | No |
| *K. pneumoniae* | NDM-71 | CP082936.1 | 48 | 2 | 0 | 0 | 1 | 1 | No | No |
| *K. pneumoniae* | R50 | CP040363.1 | 968 | 2 | 0 | 0 | 1 | 1 | No | No |
| *K. pneumoniae* | RGF99-1 | CP075552.1 | 967 | 2 | 1 | 1 | 0 | 0 | No | No |
| *K. pneumoniae* | RHB39-C10 | CP057066.1 | 161 | 2 | 0 | 0 | 1 | 1 | No | No |
| *K. pneumoniae* | S141 | CP063871.1 | 1662 | 2 | 1 | 0 | 1 | 0 | No | No |
| *K. pneumoniae* | S210-3 | CP063925.1 | 2668 | 2 | 0 | 0 | 2 | 0 | Yes | No |
| *K. pneumoniae* | WCHKP085104 | CP043362.1 | 2004 | 2 | 1 | 0 | 0 | 1 | No | No |
| *K. pneumoniae* | WCHKP13F2 | CP028391.2 | 36 | 2 | 0 | 2 | 0 | 0 | Yes | No |
| *K. pneumoniae* | ZG2017CW4-3-1-2N | CP065351.1 | 1128 | 2 | 1 | 0 | 1 | 0 | No | No |
| *K. pneumoniae* | 11219 | CP030174.1 | 1755 | 1 | 0 | 0 | 1 | 0 | Yes | No |
| *K. pneumoniae* | 12964 | CP132702.1 | 490 | 1 | 0 | 0 | 0 | 0 | Yes | No |
| *K. pneumoniae* | 13765 | JBAWHZ000000000.1 | 258 | 1 | 0 | 1 | 0 | 1 | Yes | No |
| *K. pneumoniae* | 1613163991 | CP083063.1 | 2719 | 1 | 0 | 1 | 0 | 0 | Yes | No |
| *K. pneumoniae* | 1756 | CP019219.1 | 2549 | 1 | 0 | 0 | 1 | 0 | Yes | No |
| *K. pneumoniae* | 2020CK-00223 | CP118398.1 | 491 | 1 | 0 | 1 | 0 | 0 | Yes | No |
| *K. pneumoniae* | 2021CK-01729 | CP104660.1 | 133 | 1 | 0 | 1 | 0 | 0 | Yes | No |
| *K. pneumoniae* | 33Kpn12 | CP062792.1 | 504 | 1 | 1 | 0 | 0 | 0 | Yes | No |
| *K. pneumoniae* | 463908 | CP132669.1 | 636 | 1 | 0 | 0 | 1 | 0 | Yes | No |
| *K. pneumoniae* | AR 0076 | CP032167.1 | 2398 | 1 | 0 | 0 | 0 | 0 | Yes | No |
| *K. pneumoniae* | AR8416 | CP081816.1 | 5214 | 1 | 0 | 0 | 1 | 1 | Yes | No |
| *K. pneumoniae* | ATCC 700721 | CP000647.1 | 38 | 1 | 0 | 0 | 0 | 0 | Yes | No |
| *K. pneumoniae* | Bckp091 | CP050822.1 | 18 | 1 | 0 | 0 | 1 | 1 | Yes | No |
| *K. pneumoniae* | Bckp101 | CP050840.1 | 889 | 1 | 0 | 1 | 0 | 0 | Yes | No |
| *K. pneumoniae* | C11 | CP084103.1 | 1383 | 1 | 0 | 0 | 1 | 0 | Yes | No |
| *K. pneumoniae* | CDI231 | CP077779.1 | 377 | 1 | 0 | 1 | 0 | 0 | Yes | No |
| *K. pneumoniae* | Dirty Dan | CP065436.1 | 3096 | 1 | 0 | 0 | 0 | 0 | Yes | No |
| *K. pneumoniae* | fekpn2511 | CP068972.1 | 290 | 1 | 0 | 0 | 1 | 1 | Yes | No |
| *K. pneumoniae* | GN4542 | CP102692.1 | 247 | 1 | 0 | 0 | 1 | 0 | Yes | No |
| *K. pneumoniae* | INF235 | CP031817.1 | 253 | 1 | 0 | 0 | 0 | 0 | Yes | No |
| *K. pneumoniae* | KP403 | CP089959.1 | 4845 | 1 | 0 | 1 | 0 | 1 | Yes | No |
| *K. pneumoniae* | KP53 | CP087669.1 | 37 | 1 | 0 | 1 | 0 | 0 | Yes | No |
| *K. pneumoniae* | KP85 | CP154396.1 | 11 | 1 | 0 | 0 | 0 | 0 | Yes | No |
| *K. pneumoniae* | Kp8701 | CP049604.1 | 784 | 1 | 0 | 0 | 0 | 1 | Yes | No |
| *K. pneumoniae* | KP9650 | CP122389.1 | 866 | 1 | 0 | 0 | 1 | 1 | Yes | No |
| *K. pneumoniae* | KpIM03 1 | CP076106.1 | 585 | 1 | 0 | 0 | 0 | 0 | Yes | No |
| *K. pneumoniae* | KPNIH27 | CP007731.1 | 34 | 1 | 0 | 0 | 0 | 1 | Yes | No |
| *K. pneumoniae* | KPNIH29 | CP009863.1 | 1518 | 1 | 0 | 1 | 0 | 1 | Yes | No |
| *K. pneumoniae* | LH35-d | CP100515.1 | 2053 | 1 | 0 | 0 | 0 | 0 | Yes | No |
| *K. pneumoniae* | LZSFT39 | CP132736.1 | 1306 | 1 | 1 | 0 | 0 | 1 | Yes | No |
| *K. pneumoniae* | MEI004 | CP071261.1 | 484 | 1 | 0 | 0 | 0 | 0 | Yes | No |
| *K. pneumoniae* | MV-v4-SK2-O | CP085863.1 | 5873 | 1 | 0 | 0 | 0 | 1 | Yes | No |
| *K. pneumoniae* | GCF 928375965.1 | OV754010.1 | 39 | 1 | 0 | 0 | 0 | 1 | Yes | No |
| *K. pneumoniae* | NCTC11698 | LR588408.1 | 321 | 1 | 0 | 1 | 0 | 1 | Yes | No |
| *K. pneumoniae* | PMK1 | CP008929.1 | 15 | 1 | 0 | 0 | 1 | 0 | Yes | No |
| *K. pneumoniae* | QD23 | CP042858.1 | 1 | 1 | 1 | 0 | 0 | 0 | Yes | No |
| *K. pneumoniae* | RHBSTW-00995 | CP056191.1 | 8513 | 1 | 0 | 0 | 1 | 0 | Yes | No |
| *K. pneumoniae* | RX G5M15 | CP118936.1 | 5583 | 1 | 0 | 0 | 1 | 0 | Yes | No |
| *K. pneumoniae* | ST3576 | CP103697.1 | 3576 | 1 | 0 | 0 | 0 | 0 | Yes | No |
| *K. pneumoniae* | STIN 93 | CP054972.1 | 2190 | 1 | 0 | 0 | 0 | 1 | Yes | No |
| *K. pneumoniae* | T877 | CP084241.1 | 43 | 1 | 0 | 0 | 0 | 1 | Yes | No |
| *K. pneumoniae* | TUM9839 | AP026525.1 | 1873 | 1 | 1 | 0 | 0 | 1 | Yes | No |
| *K. pneumoniae* | VA684 | CP093458.1 | 505 | 1 | 0 | 1 | 0 | 0 | Yes | No |
| *K. pneumoniae* | YZ22CS023 | CP110148.1 | 1944 | 1 | 0 | 0 | 1 | 0 | Yes | No |
| *K. pneumoniae* | YZ22CS094 | CP110167.1 | 5982 | 1 | 1 | 0 | 0 | 0 | Yes | No |
| *K. quasipneumoniae* | KqPF9 | CP065841.1 | 7051 | 19 | 4 | 3 | 9 | 3 | No | Yes |
| *K. quasipneumoniae* | OSUCMP263AKPC | CP087580.1 | 2712 | 19 | 4 | 2 | 8 | 5 | No | Yes |
| *K. quasipneumoniae* | IF1SW-B2 | CP118277.1 | 138 | 17 | 3 | 4 | 5 | 5 | No | Yes |
| *K. quasipneumoniae* | 21223 | CP091805.1 | 138 | 14 | 0 | 3 | 11 | 0 | No | Yes |
| *K. quasipneumoniae* | KqPF26 | CP065838.1 | 3387 | 13 | 2 | 1 | 3 | 7 | No | Yes |
| *K. quasipneumoniae* | M7 | CP124866.1 | 7577 | 11 | 2 | 2 | 4 | 3 | No | Yes |
| *K. quasipneumoniae* | NE1 | CP097646.1 | 841 | 10 | 1 | 3 | 3 | 3 | No | Yes |
| *K. quasipneumoniae* | SB98 | CP084803.1 | 622 | 10 | 0 | 0 | 5 | 5 | No | Yes |
| *K. quasipneumoniae* | WP7-S17-ESBL-08 | AP022183.1 | 1031 | 9 | 2 | 0 | 2 | 5 | No | No |
| *K. quasipneumoniae* | KW1 | CP102898.1 | 526 | 8 | 0 | 1 | 3 | 4 | No | No |
| *K. quasipneumoniae* | D120-1 | CP034678.1 | 1770 | 7 | 3 | 1 | 3 | 0 | No | No |
| *K. quasipneumoniae* | FDAARGOS 1504 | CP083631.1 | 1215 | 6 | 2 | 2 | 1 | 1 | No | Yes |
| *K. quasipneumoniae* | 09A323 | CP084783.1 | 414 | 6 | 2 | 0 | 2 | 2 | No | No |
| *K. quasipneumoniae* | 12A476 | CP084779.1 | 384 | 6 | 2 | 2 | 1 | 1 | No | No |
| *K. quasipneumoniae* | 3Z-5L | CP072515.1 | 3830 | 6 | 3 | 1 | 1 | 1 | No | No |
| *K. quasipneumoniae* | Pinhead Larry | CP065467.1 | 9043 | 6 | 0 | 2 | 1 | 3 | No | No |
| *K. quasipneumoniae* | FDAARGOS 1443 | CP077279.1 | 2275 | 5 | 2 | 0 | 3 | 0 | No | No |
| *K. quasipneumoniae* | MMCC7 | CP082785.1 | 5013 | 5 | 0 | 1 | 3 | 1 | No | No |
| *K. quasipneumoniae* | KqPF42 | CP065846.1 | 1535 | 4 | 1 | 1 | 0 | 2 | No | Yes |
| *K. quasipneumoniae* | 2022CK-00516 | CP114160.1 | 3890 | 4 | 1 | 0 | 3 | 0 | No | No |
| *K. quasipneumoniae* | IIIF3SW-P1 | CP118278.1 | 3234 | 4 | 1 | 0 | 2 | 1 | No | No |
| *K. quasipneumoniae* | KP18-31 | CP045641.1 | 852 | 4 | 1 | 1 | 2 | 0 | No | No |
| *K. quasipneumoniae* | NCTC9170 | LR588411.1 | 488 | 3 | 0 | 0 | 2 | 1 | No | Yes |
| *K. quasipneumoniae* | ARLG-8054 | CP067966.1 | 6953 | 3 | 0 | 1 | 1 | 1 | No | No |
| *K. quasipneumoniae* | G747 | CP034136.1 | 476 | 3 | 0 | 1 | 1 | 1 | No | No |
| *K. quasipneumoniae* | HKUOPA4 | CP014154.1 | 3870 | 3 | 0 | 2 | 0 | 1 | No | No |
| *K. quasipneumoniae* | NDM-101 | CP085197.1 | 138 | 3 | 1 | 0 | 0 | 2 | No | No |
| *K. quasipneumoniae* | 203 19001 | CP062138.1 | 9546 | 2 | 0 | 0 | 0 | 2 | Yes | No |
| *K. quasipneumoniae* | 68-17 | CP042319.1 | 3540 | 2 | 1 | 0 | 0 | 1 | No | No |
| *K. quasipneumoniae* | KA1 | CP102893.1 | 526 | 2 | 1 | 1 | 0 | 0 | No | No |
| *K. quasipneumoniae* | KP20-558 | CP096263.1 | 978 | 2 | 0 | 0 | 2 | 0 | Yes | No |
| *K. quasipneumoniae* | KQ20605 | CP133205.1 | 2558 | 2 | 0 | 1 | 0 | 1 | No | No |
| *K. quasipneumoniae* | GCF 905331345.2 | NZ_OW968431.1 | 6862 | 2 | 0 | 1 | 0 | 1 | No | No |
| *K. quasipneumoniae* | SB1124 | CP084806.1 | 2274 | 2 | 1 | 1 | 0 | 0 | No | No |
| *K. quasipneumoniae* | SB610 | CP084770.1 | 2276 | 2 | 0 | 0 | 0 | 2 | Yes | No |
| *K. quasipneumoniae* | TH114 | CP035207.1 | 1321 | 2 | 1 | 0 | 0 | 1 | No | No |
| *K. quasipneumoniae* | Y7-3 | CP099834.1 | 9297 | 2 | 0 | 0 | 0 | 2 | Yes | No |
| *K. quasipneumoniae* | SWHEFF 72 | CP055010.1 | 334 | 1 | 0 | 0 | 1 | 0 | Yes | Yes |
| *K. quasipneumoniae* | na | CP014696.2 | 489 | 1 | 0 | 1 | 0 | 0 | Yes | No |
| *K. quasipneumoniae* | 01A030T | CP084876.1 | 1528 | 1 | 1 | 0 | 0 | 0 | Yes | No |
| *K. quasipneumoniae* | 18A069 | CP084818.1 | 1118 | 1 | 1 | 0 | 0 | 0 | Yes | No |
| *K. quasipneumoniae* | AR8538 | CP081827.1 | 5820 | 1 | 1 | 0 | 0 | 0 | Yes | No |
| *K. quasipneumoniae* | BS418 | CP063942.1 | 9669 | 1 | 0 | 0 | 0 | 0 | Yes | No |
| *K. quasipneumoniae* | EF262 | CP092512.1 | 5015 | 1 | 0 | 1 | 0 | 1 | Yes | No |
| *K. quasipneumoniae* | FDAARGOS 93 | CP014071.1 | 489 | 1 | 0 | 0 | 1 | 0 | Yes | No |
| *K. quasipneumoniae* | KPC142 | CP023478.1 | 3874 | 1 | 0 | 0 | 1 | 0 | Yes | No |
| *K. quasipneumoniae* | NCTC11357 | LR134196.1 | 1226 | 1 | 0 | 1 | 0 | 0 | Yes | No |
| *K. quasipneumoniae* | WI-06 | CP081893.1 | 7119 | 1 | 0 | 0 | 1 | 0 | Yes | No |
| *K. quasipneumoniae* | YDKL-002 | CP068237.1 | 9670 | 1 | 0 | 0 | 0 | 1 | Yes | No |
| *K. variicola* | M186-1 | CP063911.1 | 9395 | 14 | 3 | 1 | 4 | 6 | No | Yes |
| *K. variicola* | BS326-1 | CP063898.1 | 347 | 11 | 2 | 3 | 2 | 4 | No | No |
| *K. variicola* | NCTC9668 | LR134235.1 | 3573 | 10 | 2 | 0 | 2 | 6 | No | Yes |
| *K. variicola* | 04153260899A | LR130543.1 | 355 | 9 | 2 | 0 | 3 | 4 | No | No |
| *K. variicola* | AJ055 | LR130539.1 | 695 | 8 | 1 | 1 | 4 | 2 | No | Yes |
| *K. variicola* | INF352 | CP083790.1 | 347 | 8 | 1 | 2 | 3 | 2 | No | No |
| *K. variicola* | 13450 | CP026013.1 | 1562 | 6 | 1 | 1 | 2 | 2 | No | No |
| *K. variicola* | E57-7 | CP016344.1 | 697 | 5 | 2 | 2 | 1 | 0 | No | No |
| *K. variicola* | KPN1481 | CP020847.1 | 906 | 5 | 1 | 0 | 2 | 2 | No | No |
| *K. variicola* | 15WZ-82 | CP032354.1 | 595 | 4 | 1 | 1 | 1 | 1 | No | Yes |
| *K. variicola* | 17YN56 | CP097808.1 | 197 | 4 | 0 | 1 | 3 | 0 | No | No |
| *K. variicola* | GN02 | CP031061.1 | 5059 | 4 | 2 | 0 | 1 | 1 | No | No |
| *K. variicola* | WCHKV030666 | CP027064.2 | 595 | 4 | 1 | 0 | 0 | 3 | No | No |
| *K. variicola* | 01A065 | CP084766.1 | 2273 | 3 | 0 | 0 | 0 | 3 | Yes | No |
| *K. variicola* | E25 | CP067261.1 | 3523 | 3 | 0 | 0 | 1 | 2 | No | No |
| *K. variicola* | F2R9T | CP084765.1 | 2263 | 3 | 0 | 2 | 1 | 0 | No | No |
| *K. variicola* | M142-3 | CP063867.1 | 3972 | 3 | 1 | 0 | 1 | 1 | No | No |
| *K. variicola* | BS359-2-1 | CP063912.1 | 919 | 2 | 1 | 0 | 0 | 1 | No | No |
| *K. variicola* | JCM 12419 | AP024592.1 | 1169 | 2 | 0 | 0 | 0 | 2 | Yes | No |
| *K. variicola* | KGB 1 | CP071734.1 | 3531 | 2 | 1 | 0 | 0 | 1 | No | No |
| *K. variicola* | KPN692 | CP089385.1 | 5823 | 2 | 1 | 1 | 0 | 0 | No | No |
| *K. variicola* | ML 9ba2 | CP092632.1 | 347 | 2 | 0 | 0 | 1 | 1 | No | No |
| *K. variicola* | na | NZ_OW969912.1 | 2295 | 2 | 0 | 0 | 0 | 2 | Yes | No |
| *K. variicola* | AHKv-S01 | CP047360.1 | 9392 | 1 | 0 | 0 | 1 | 0 | Yes | No |
| *K. variicola* | AJ292 | CP149491.1 | 2122 | 1 | 0 | 1 | 0 | 0 | Yes | No |
| *K. variicola* | At-22 | CP001891.1 | 1220 | 1 | 0 | 0 | 0 | 1 | Yes | No |
| *K. variicola* | FF907 | CP077805.1 | 5032 | 1 | 0 | 0 | 0 | 1 | Yes | No |
| *K. variicola* | J57 | CP124804.1 | 971 | 1 | 0 | 1 | 0 | 0 | Yes | No |
| *K. variicola* | KV4 | CP103419.1 | 3948 | 1 | 0 | 0 | 0 | 1 | Yes | No |
| *K. variicola* | SY1 | CP106964.1 | 357 | 1 | 0 | 0 | 0 | 1 | Yes | No |
| Total |  |  |  | 937 | 179 | 169 | 349 | 240 |  |  |

* Indicates isolates that were only found in one age or hospitalized subgroup

** Indicates isolates that were associated with carriage events (i.e., found in more than one consecutive timepoint for an individual)

**Table S7. Correlation data for age-antibody level comparisons**

|  | years vs. O1 | years vs. O2 | years vs. O3 | years vs. O5 | years vs. K1 | years vs. K2 | years vs. K107 |
| --- | --- | --- | --- | --- | --- | --- | --- |
| **Pearson r** |  |  |  |  |  |  |  |
| r | 0.5326 | 0.4473 | 0.6016 | 0.4835 | 0.4696 | 0.5435 | 0.5419 |
| 95% confidence interval | 0.3544 to 0.6735 | 0.2524 to 0.6074 | 0.4400 to 0.7254 | 0.2952 to 0.6357 | 0.2787 to 0.6249 | 0.3677 to 0.6818 | 0.3658 to 0.6806 |
| R squared | 0.2837 | 0.2001 | 0.3619 | 0.2338 | 0.2206 | 0.2954 | 0.2937 |
|  |  |  |  |  |  |  |  |
| **P value** |  |  |  |  |  |  |  |
| P (two-tailed) | <0.0001 | <0.0001 | <0.0001 | <0.0001 | <0.0001 | <0.0001 | <0.0001 |
| P value summary | **** | **** | **** | **** | **** | **** | **** |
| Significant? (alpha = 0.05) | Yes | Yes | Yes | Yes | Yes | Yes | Yes |
|  |  |  |  |  |  |  |  |
| **Number of XY Pairs** | 80 | 80 | 80 | 80 | 80 | 80 | 80 |

**Table S8. Demographic and Clinical Metadata.** (excel table)

**Table S9. NCBI BioSample accession numbers** (excel table)
